# Efficient calculation of exact probability distributions of integer features on RNA secondary structures

**DOI:** 10.1186/1471-2164-15-S10-S6

**Published:** 2014-12-12

**Authors:** Ryota Mori, Michiaki Hamada, Kiyoshi Asai

**Affiliations:** 1 Graduate School of Frontier Sciences, The University of Tokyo, Kashiwa City, Chiba, Japan; 2 Computational Biology Research Center, National Institute of Advanced Industrial Science and Technology (AIST), Koto Ward, Tokyo, Japan; 3 Faculty of Science and Engineering, Waseda University, Shinjuku Ward, Tokyo, Japan

**Keywords:** RNA secondary structure, integer score distribution, structural landscape

## Abstract

**Background:**

Although the needs for analyses of secondary structures of RNAs are increasing, prediction of the secondary structures of RNAs are not always reliable. Because an RNA may have a complicated energy landscape, comprehensive representations of the whole ensemble of the secondary structures, such as the probability distributions of various features of RNA secondary structures are required.

**Results:**

A general method to efficiently compute the distribution of any integer scalar/vector function on the secondary structure is proposed. We also show two concrete algorithms, for Hamming distance from a reference structure and for 5ʹ − 3ʹ distance, which can be constructed by following our general method. These practical applications of this method show the effectiveness of the proposed method.

**Conclusions:**

The proposed method provides a clear and comprehensive procedure to construct algorithms for distributions of various integer features. In addition, distributions of integer vectors, that is a combination of different integer scores, can be also described by applying our 2D expanding technique.

## Background

Recent investigations of coding and non-coding RNAs have proved that RNA molecules have more important roles in the regulation of living cells than those of our previous knowledge. It has also become clear that the structures of RNAs, especially the secondary structures, are one of the important features to identify the functions of RNAs. While the high-throughput methods to determine the secondary structures of RNAs are spreading, the importance of computational analyses of RNA sequences including prediction of secondary structures is increasing [1,2].

The free energy of each structure is connected to its existence probability. The existence probability of a secondary structure St of an RNA is given by the following canonical distribution:

(1) $$P_{\text{St}}=\frac{1}{Z}e^{-E_{\text{St}}\text{/}\left(k_{B}T\right)}$$

(2) $$Z=\sum_{\text{St}}e^{-E_{\text{St}}\text{/}(k_{B}T)},$$

where *P*_St _and *E*_St _are respectively the existence probability and the free energy of the structure St, *k_B _*is the Boltzmann constant and *T *is the temperature constant. *Z *is the normalizing factor known as the partition function, which is the summation of Boltzmann factor *e*^−*E*_St _/(*k*_*B*_*T*) ^among all the possible structures. The partition function of an RNA sequence and the free energy of each structure can be obtained by dynamic programming algorithms on the parameters determined experimentally [3,4].

The equation (1) shows that the structure with the highest existence probability is the structure of the minimum free energy. Therefore, it is natural to treat the secondary structure of the minimum free energy as the estimate of the secondary structure. The probability that an RNA folds into a particular structure is, however, generally extremely low even if it is the structure of the minimum free energy, because of the combinatorial explosion [5]. For example, the probability of a particular secondary structure of some rRNAs are less than 10^−22 ^no matter which structure is chosen. This means that the prediction of the secondary structure of an RNA and subsequent analyses based on the predicted secondary structure are not always reliable. It is therefore desirable to investigate properties of the probability distributions of the whole ensemble of the possible structures.

We propose in this paper a general method to efficiently compute the exact distribution of any integer quantity of the feature on each secondary structure. The proposed method has been motivated by the framework and its application to sequence alignments by [6]. Their framework is generally valid for integer functions on Boltzmann distributions whose partition function can be calculated by a linear dynamic programming. For the case of secondary structures of RNAs, however, the recursions in the dynamic programming of the partition function have more complicated forms including the products of combinations of DP matrix elements, which inhibits direct application of their framework. We have overcome the difficulty by expanding McCaskill algorithm, which is a well-known dynamic programming of the partition function of the secondary structures of RNAs using the energy parameters experimentally determined [7].

Naive implementations of our proposed method requires computational complexities of $O\left(n^{3}|S{|}^{2}\alpha \right)$ in time and $O\left(n^{3}+\beta |S|\right)$ in space, where *n *is the length of the sequence, |*S*| is the size of the integer score variation, which depends on the objective distribution while they never exceed *n *in the case of example problems in this paper, and *α *and *β *is the costs depending on the objective score. By adapting Discrete Fourier Transform, we can reduce those complexities to $O\left(n^{3}|S|\alpha \right)$ in time and $O\left(n^{3}+\beta \right)$. The DFT in our method on RNA structures achieves an order-level improvement of the complexity, which could not achieved by the DFT on linear dynamic programmings in [6]. We can further reduce time complexity to $O\left(n^{3}|S|\alpha \text{/}U\right)$ by parallel computing using *U *computational units.

We demonstrate the effectiveness of the proposed method in several practical problems. The first example is the distribution of the Hamming distances from a reference structure. A practically equivalent algorithm and its acceleration have been implemented as RNAbor by [8] and [9], while we have reconstructed the algorithm by deducing from our general principle. The second example is the exact distributions of 5ʹ - 3ʹ distance. Conventional methods for analysing 5ʹ - 3ʹ distance only calculate mean length or assume over-simplified models. We propose here a novel algorithm to compute the whole distribution of 5ʹ - 3ʹ distance considering the thermodynamic properties of the RNAs. The final example is acceleration of RNA2Dfold, which is included in ViennaRNA package [10].In this example, the distribution of the Hamming distances from two specified reference structures are calculated. We show our method reduces computational complexity from *O*(*n*^7^) in time and *O*(*n*^4^) in re-space to less than *O*(*n*^5^*/U *) in time and *O*(*n*^2^*U *) in space, which is a similar idea proposed recently [11]. These examples indicate that our method offers a way to obtain a wide variety of distributions of integer quantities.

## Methods

We first show the fundamental concepts of our proposed method in this section.

### Definition of integer score distribution

Let us assume that *s *represents a mapping from *x *∈ *U *to an integer score s(x)∈ℤ. In our case of RNA secondary structures, the *U *is the space of all the possible secondary structures for a given RNA sequence, and an integer score *s*(*x*) represents a feature or a property assigned to each structure *x*. The integer score distribution is defined as the probability distribution *p*(*s*) of *s*(*x*) derived from the probability distribution *p*(*x*) of *x*:

(3) $$p\left(s\right)=\underset{\left\{x|s=s\left(x\right)\right\}}{\sum }p\left(x\right)$$

In this paper, we discuss on how to efficiently compute integer score distributions in general and in the specific cases for RNA secondary structures. Our proposed method for RNA secondary structures efficiently computes the exact distribution when *p*(*x*) and *p*(*s*) can be calculated by the dynamic programming algorithms sharing a same form.

### A conventional model for integer score distribution

For a certain class of problems, including distributions of integer score of each sequence alignment, the partition function of the objective distribution can be calculated abstractly by Algorithm 1. *Z *is the partition function shown in equation (2). *Z *is a scalar array of length *N *representing the partition function of the problem size *N *, whose components for the dynamic programming are aligned in the computing order. *t*(*k|i*) is a quantity proportional to the probability of the transition from state *i *to state *k*, which can be quite sparse in values.

**Algorithm 1 **An abstract form of calculating the partition function

1: *Z*[0] = 1

2: **for ***k *= 1 to *N ***do**

3:    $z[k]={\text{Σ}}_{i=0}^{k-1}Z[i]t\left(k\text{|}i\right)$

4: **end for**

5: *Z *= *Z*[*N *]

[6] showed that if the partition function can be computed by Algorithm 1, integer score distributions are obtained by Algorithm 2, where *Z*(*x*) is an array of polynomials of *x*, and *s*(*i, k*) is the gain of the integer score in the transition from *i *to *k*.

**Algorithm 2 **A polynomial approach to integer score distributions proposed in [6]

1: *Z*(*x*)[0] = 1

2: **for ***k *= 1 to *N ***do**

3:    $Z\left(x\right)[k]={\text{Σ}}_{i=0}^{k-1}Z\left(x\right)[i]t\left(k\text{|}i\right)x^{s\left(i,k\right)}$

4: **end for**

5: *Z *= a sum of coefficients of polynomial *Z*(*x*)[*N *]

In Algorithm 2, *Z*(*x*)[*N*] represents a polynomial in *x *whose factor *z_S _*of *x^i ^*is proportional to the sum of the probabilities of obtaining score *i *among all the paths:

(4) $$Z\left(x\right)\left[N\right]=\overset{S_{max}}{\underset{j=0}{\sum }}z_{j}x^{j},$$

where *S_max _*is the maximum score.

The *p_S_*, the probability of obtaining score *S*, is finally calculated by the following equation:

(5) $$p_{S}=\frac{z_{S}}{Z},$$

### A general model for integer score distribution of RNA secondary structure

In the case of RNA secondary structures, the dynamic programming for the partition function does not match to Algorithm 1. Therefore, we have to construct an algorithm different from Algorithm 2 for the calculation of integer score distributions on RNA secondary structures. As pseudo-code is shown in Algorithm 3, products of combinations between DP matrix elements and constant term *c_k _*are required for the computation. The detailed description of this derivation is shown in the additional file 1 (Section S1).

**Algorithm 3 **A general polynomial approach to integer score distributions for the ensemble of RNA secondary structures

1: *Z*(*x*)[0] = 1

2: **for ***k *= 1 to *N ***do**

3:    $Z\left(x\right)[k]={\text{Σ}}_{i=0}^{k-1}Z\left(x\right)[i]t\left(k\text{|}i\right)x^{s\left(i,k\right)}+$${\text{Σ}}_{i=0}^{k-2}{\text{Σ}}_{j=i+1}^{k-1}Z\left(x\right)\left[i\right]Z\left(x\right)\left[j\right]t\left(k\text{|}i,j\right)x^{s\left(i,j,k\right)}+c_{k}x^{s\left(k\right)}$

4: **end for**

5: *Z *= a sum of coefficients of polynomial *Z*(*x*)[*N *]

The partition function is dispersed according to the score of each secondary structure included in the whole ensemble. In other words, the coefficient of *x^S ^*in *Z*(*x*)[*N*] represents proportional to the probability that the RNA structure has score *S*. After the calculation by Algorithm 3, *p_S _*can be derived from equation (5).

Algorithm 3 requires computational complexities of $O\left(n^{3}S_{max}^{2}\alpha \right)$ in time and $O\left(\left(n^{2}+\beta \right)S_{max}\right)$ in memory, where *α *and *β *is the complexities in time and in space respectively for the calculation of each integer score.

### Adopting Discrete Fourier Transform (DFT)

Discrete Fourier Transform (DFT) is a Fourier Transform on a discrete sampling interval, which is employed in improving the efficiency of various computational problems as well as frequency analysis. According to [6], by applying DFT distributed processing is available for computing integer score distributions on sequence alignments. On RNA secondary structures, DFT reduces time complexity of computations in order-level as well as merely decentralize the procedure.

DFT  F satisfies the following equation:

(6) z=F(ζ),

where

(7) $$z=\left(z_{0},z_{1},\cdots \;,z_{S_{max}}\right)$$

(8) $$\zeta =\left(\zeta_{0},\zeta_{1},\cdots \;,\zeta_{S_{max}}\right)$$

(9) $$\zeta_{k}=\frac{{\text{Σ}}_{j=0}^{S_{max}}\;z_{j}{\left(\text{exp}\left[2\pi i\frac{k}{S_{max}+1}\right]\right)}^{j}}{S_{max}+1}.$$

In DFT approach, each *x *in the polynomials is replaced by a complex number on the unit circle to calculate ***ζ ***instead of **z **directly. The relation of the two quantities are derived by comparing equations (4) and (9)):

(10) $$\zeta_{k}=\frac{Z\left(\text{exp}\left[2\pi i\frac{k}{S_{max}+1}\right]\right)\left[N\right]}{S_{max}+1}.$$

After ***ζ ***is obtained, DFT extracts **z **from ***ζ ***by $O\left(S_{max}^{2}\right)$ time.

Algorithm 4 shown below is the modification of our naive Algorithm 3 by adopting DFT approach.

Algorithm 3 suffers from heavy computations of $O\left(S_{max}^{2}\right)$ in time for products of polynomials if the degree *S_max _*is large. In the recursions for ***ζ ***in Algorithm 4, however, each computation for polynomial products is replaced to a computation of products of complex numbers, which requires only a constant time. While we still need to extract **z **from ***ζ ***by $O\left(S_{max}^{2}\alpha \right)$ time, the total time complexity is reduced from $O\left(n^{3}S_{max}^{2}\alpha \right)$ to $O\left(n^{3}S_{max}^{2}\alpha \right)$. In addition, each *ζ_k _*can be calculated

**Algorithm 4 **DFT-adopted approach for integer score distribution

1: /* DP phase (distributed processing is available) */

2: **for ***S *= 0 to *S_max _***do**

3:    $x=\text{exp}\left[2\pi i\frac{S}{S_{max}+1}\right]$

4:    *Z*[*S*][0] = 1

5:    **for ***k *= 1 to *N ***do**

6:    $Z[S]\left[k\right]={\text{Σ}}_{p=0}^{k-1}Z\left[S\right]\left[p\right]t\left(k\text{|}p\right)x^{s\left(p,k\right)}+$${\text{Σ}}_{p=0}^{k-2}{\text{Σ}}_{q=p+1}^{k-1}Z\left[S\right]\left[p\right]Z\left[S\right]\left[q\right]t\left(k\text{|}p,q\right)x^{s\left(p,q,k\right)}+c_{k}x^{s\left(k\right)}$

7:    **end for**

8:    *ζ_S _*= *Z*[*S*][*N*]

9: **end for**

10: /* DFT phase*/

11: **for ***S *= 0 to *S_max _***do**

12:    $z_{S}={\text{Σ}}_{r=0}^{S_{max}}\zeta_{r}\text{exp}\left[-2\pi i\frac{rS}{S_{max}+1}\right]/\left(1+S_{max}\right)$

13: **end for**

14: $Z={\text{Σ}}_{S=0}^{S_{max}}z_{S}$

individually so we can replace the computational cost to $O\left(n^{3}\alpha \right)$ time and $O\left(\left(n^{2}+\beta \right)S_{max}\right)$ space by adopting maximum parallelization, using ether multi-core units or cluster machines. Accordingly, the practical efficiency by utilizing DFT depends on parallelization environment strongly (Table 1).

**Table 1:** Required time and space

	Polynomial	DFT	DFT with U* units
Time	$O\left(n^{3}{S^{2}}_{max}\alpha \right)$	$O\left(n^{3}S_{max}\;\alpha \right)$	$O\left(n^{3}S_{max}\alpha /U\right)$
Space	$O\left((n^{2}+\beta )S_{max}\right)$	$O\left(n^{2}+\beta \right)$	$O\left(\left(n^{2}+\beta \right)U\right)$

*^∗^*The number of parallelization units must be equal to *S_max _*or less. If *U *= *S_max_*, DFT with *U *units requires *O*(*n*^3^*α*) in time and $O\left(\left(n^{2}+\beta \right)S_{max}\right)$ in space.

### McCaskill model

According to the above approach, we next construct and implement concrete formulas of computing a general integer score distribution for RNA secondary structures based on McCaskill model. McCaskill model is a standard procedure for computing partition function in equation (2) by a dynamic programming based on energy parameters. In this model, the partition function is obtained as *Z*_1,*n *_from the following recursive scheme of polynomial order:

**Initialization **(1 ≤ *i *≤ *n*):

(11) $$Z_{i,i}=1.0$$

(12) $$Z_{i,i}^{∙}=Z_{i,i-1}^{m}=0,$$

**Recursion **(1 ≤ *i *≤ *j *≤ *n*):

(13) $$Z_{i,j}=1.0+\overset{j-1}{\underset{k=i}{\sum }}Z_{i,k}Z_{k+1,j}^{1}$$

(14) $$Z_{i,j}^{1}=\overset{j}{\underset{k=i+1}{\sum }}Z_{i,k}^{b}$$

(15) $$\begin{array}{c} Z_{i,j}^{b}=e^{f_{1}\left(i,j\right)}+\overset{j-2}{\underset{k=i+1}{\sum }}\overset{j-1}{\underset{l=k+1}{\sum }}Z_{k,l}^{b}e^{f_{2}\left(i,j,k,l\right)}\\ \;+\overset{j-1}{\underset{k=i+2}{\sum }}Z_{i+1,k-1}^{m}Z_{k,j-1}^{m1}e^{f_{3}\left(i,j\right)} \end{array}$$

(16) $$Z_{i,j}^{m}=\overset{j-1}{\underset{k=i}{\sum }}\left(e^{f_{4}\left(k-i\right)}+Z_{i,k-1}^{m}\right)Z_{k,j}^{m1}$$

(17) $$Z_{i,j}^{m1}=\overset{j}{\underset{k=i+1}{\sum }}Z_{i,k}^{b}e^{f_{4}\left(j-k\right)},$$

where each *f_k _*(*·*) (*k *= 1 *· · · *4) is the function corresponding to the energy contribution to each state, and the parameters of the functions are determined experimentally [3,4].

(18) $$f_{k}\left(\cdot \right)=-\frac{\Delta E}{k_{B}T}$$

Although the second factor in the right hand side of the equation (15) indicates that this procedure requires *O*(*n*^4^) in time, it is usually reduced to *O*(*n*^3^) by assuming a reasonable threshold of the length of the internal loops.

### Score accumulable McCaskill model

We modify McCaskill model recursions (equations (13)-(17)) to calculate integer score distribution under the concept described in the Approach section.

(19) $$Z_{i,j}=x^{g_{1}(i,j)}+\sum_{k=i}^{j-1}Z_{i,k}Z_{k+1,j}^{1}x^{g_{2}(i,j,k)}$$

(20) $$Z_{i,j}^{1}=\overset{j}{\underset{k=i+1}{\sum }}Z_{i,k}^{b}x^{g_{3}\left(i,j,k\right)}$$

(21) $$\begin{array}{c} Z_{i,j}^{b}=e^{f_{1}\left(i,j\right)}x^{g_{4}\left(i,j\right)}\\ \;\;\;+\overset{j-2}{\underset{k=i+1}{\sum }}\overset{j-1}{\underset{l=k+1}{\sum }}Z_{k,l}^{b}e^{f_{2}\left(i,j,k,l\right)}x^{g_{5}\left(i,j,k,l\right)}\\ \;\;\;+\overset{j-1}{\underset{k=i+2}{\sum }}Z_{i+1,k-1}^{m}Z_{k,j-1}^{m1}e^{f_{3}\left(i,j\right)}x^{g_{6}\left(i,j,k\right)} \end{array}$$

(22) $$Z_{i,j}^{m}=\overset{j-1}{\underset{k=i}{\sum }}\left(e^{f_{4}\left(k-i\right)}x^{g_{7}\left(i,j,k\right)}+Z_{i,k-1}^{m}x^{g_{8}\left(i,j,k\right)}\right)Z_{k,j}^{m1}$$

(23) $$Z_{i,j}^{m1}=\overset{j}{\underset{k=i+1}{\sum }}Z_{i,k}^{b}e^{f_{4}\left(j-k\right)}x^{g_{9}\left(i,j,k\right)},$$

## Results

In this section, we show three examples to demonstrate how to construct algorithms for practical problems. The first and the second examples are the case to which our general model is directly applicable, where all we have to do is defining scoring functions. In the third example, we expand our model into two dimensions in order to describe a distribution of two dimensional integer vector.

We practically implemented and evaluated the concrete algorithms for those three examples with a distributed processing application by OpenMP on a dual quad-core Intel^® ^Xeon^® ^E5540 @2.53GHz with 17.6 GB RAM. The run time was measured as a mean of 10 random sequences by single or eight cores.

### A distribution of the Hamming distance from a reference structure

Conventional RNA secondary structure estimation produces the most stable and possible structure or the representative structure such as a centroid in the whole ensemble. Those point estimations of the secondary structures, however, have a risk to neglect the information on the thermal fluctuations or significant suboptimal structures [12]. Some local structures might be relatively stable only at certain global structures, and some structures such as ribo-switches might have multiple distinct stable global structures besides the predicted structures [13]. RNAbor [8] is a software which exactly calculates the probability that RNA folds into the structures that have the same distance from a given structure. It informs us concentration of existence probability around a structure predicted by conventional software, which will help deeper understanding about biological behavior of RNA molecules. Our model is applicable for this problem since the distance between RNA secondary structures can be defined as an integer function. Here we reconstruct the algorithm from a viewpoint of our general principle described in the Approach section, motivated by the work by Newberg *et al*., while practically equivalent algorithm has been independently presented in [9].

#### Definition of distance

We employ the distance measure of RNA secondary structures used in RNAbor, which is defined as the Hamming distance between binary vectors representing the structures as described below.

(24) $$S\left[i\right]\left[j\right]=\left\{\begin{array}{cc} 1 & \left(\text{if i - th and j - th bases make a pair}\right)\\ 0 & \left(\text{otherwise}\right) \end{array}.\right)$$

Let us call *S *a structure vector. The dimension of a structure vector is $\left(\begin{array}{c} n\\ 2 \end{array}\right)=n\left(n-1\right)/2$ for an RNA of length *n*.

Now we define the Hamming distance *d *of two structures by the Hamming distance of their structure vectors *S*_1 _and *S*_2_:

$$\begin{array}{c} d=\overset{n-1}{\underset{i=1}{\sum }}\overset{n}{\underset{j=i+1}{\sum }}S_{1}\left[i\right]\left[j\right]⊕S_{2}\left[i\right]\left[j\right]\\ \;\;\;⊕\;:\text{exclusive disjunction}\text{.} \end{array}$$

The Hamming distance between RNA structures never exceeds its sequence length *n *in spite of the high dimensions of structure vectors, we obtain *d_max _*≤ *n *as the exact maximum of *d *by cubic time (See the Section S3 in the additional file 1).

#### Scoring functions

Recursions for calculating the distribution of *d *are easily derived by defining *gk *(*·*) (*k *= 1 *· · · *9) in the equations (19)-(23) as appropriate integer functions. For instance:

(26) $$\begin{array}{c} g_{6}\left(i,j,k\right)=\overset{j-1}{\underset{p=k}{\sum }}S\left[p\right]\left[j\right]+\overset{j}{\underset{q=i+1}{\sum }}S\left[i\right]\left[q\right]\\ \;\;\;\;+\overset{k-1}{\underset{p=i+1}{\sum }}\overset{j}{\underset{q=k}{\sum }}S\left[p\right]\left[q\right]+1-2S\left[i\right]\left[j\right] \end{array}$$

**Figure 1:**
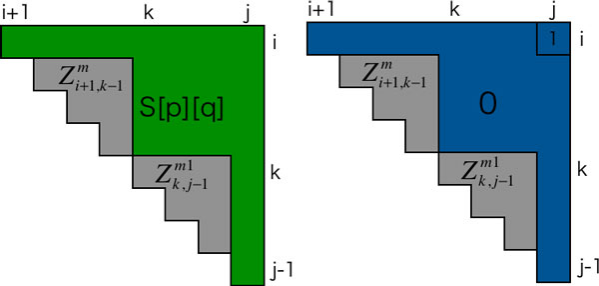
**A simple concept illustration of the way to calculate the newly accumulated distance**. Left and right pictures illustrate the vector of the reference structure and the transition which corresponds to the third term on right side of equation (21) respectively. *g*_6_(*i, j, k*) returns the Hamming distance between green and blue regions. Gray regions have been already considered in $Z_{i+1,k-1}^{m}$ and $Z_{k,j-1}^{m1}.$

This *g*_6_(·) returns an integer value that is newly accumulated as the gain of the Hamming distance from the reference structure by the corresponding transition (Figure 1). Although naive implementation for computation of *g_k_*(·) requires quadratic order in time, a slight pre-calculation reduces this to constant time. We show full description of *g_k_*(·) and *O*(1) time calculation in the additional file 1 (Section S2). Accordingly, the total complexity using DFT is $O\left(n^{3}d_{max}\right)$ in time and *O*(*n*^2^) in space, since *S_max _*= *d_max_, α *= 1, and *β *= *O*(*n*^2^). It can be reduced to $O\left(n^{3}d_{max}\text{/}U\right)$ in time and *O*(*n*^2^*U *) in space if parallelization of *U-*units is available(*U *≤ *d_max_*).

### A distribution of RNA 5ʹ- 3ʹdistance

Recently, Yoffe *et al*. found that the distance of 5ʹ end and 3ʹ end of the RNA molecule tended to be short, largely independent of molecule lengths or sequence patterns [14]. They pointed out the relevance of these observations and biological interpretation especially about in viral RNA evolution. Clote *et al*. proposed a method for calculating an expected distance [15], but it might be helpful for RNA structure analysis to reveal the population of structures shorter than some threshold as well as mean length. A method for counting the 5ʹ-3ʹ distances over all secondary structures has been proposed by [16], but their method assumes that all structures occur by the same probability and every base can make pairs with an arbitrary base except for pseudoknots. We propose the first algorithm for computing the exact probability distribution of the 5ʹ-3ʹ distances based on the McCaskill model.

#### Definition of 5ʹ - 3ʹ distance

**Figure 2:**
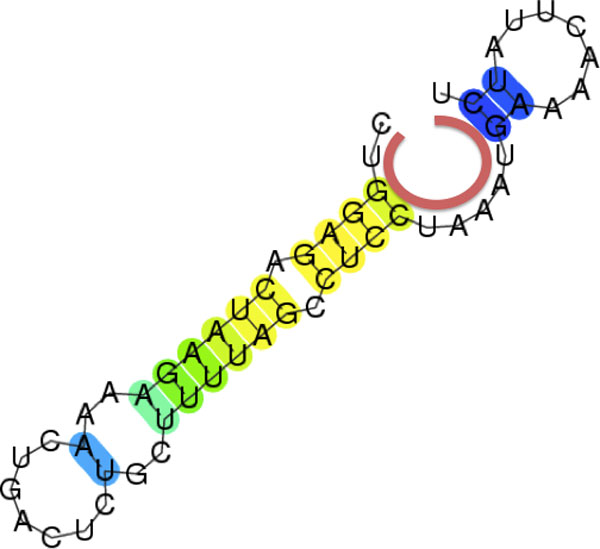
**An example for introducing the definition of *d*5ʹ - 3ʹ**. The red arch represents the 5ʹ - 3ʹ distance, in this case, we have *d*_5ʹ *- *3ʹ _= *c_ext _*+ *h_ext _*= 3 + 8 = 11.

We follow the work by Yoffe and colleagues as the definition of 5ʹ - 3ʹ distance $d_{5\prime -3\prime }$:

(27) $$d_{5\prime -3\prime }=c_{ext}+h_{ext},$$

where *c_ext _*is the number of covalent bonds in the exterior loop and *h_ext _*is the number of hydrogen bridges in the exterior loop (See Figure 2 for example).

#### Scoring functions

As with the case of the previous section, defining *gm*(·) (*m *= 1 · · · 9) enables us to calculate the 5ʹ-3ʹ distance distribution as following:

(28) $$g_{1}\left(i,j\right)=j-i$$

(29) $$g_{2}\left(i,j,k\right)=1$$

(30) $$g_{3}\left(i,j,k\right)=1+j-k$$

(31) $$g_{m}\left(\cdot \right)=0\;\left(\text{for}\;m=4,\ldots ,9\right)$$

The *g*_1_(*i, j*) is the 5ʹ - 3ʹ distance of the chain structure, which contains no base pairs. The *g*_2_(*i, j, k*) is the newly accumulated 5ʹ - 3ʹ distance, that is the junction of *k*-th and *k*+1-th bases. The *g*_3_(*i, j, k*) represents the sum of a hydrogen bridge by *i*-th and *k*-th bases and length of a chain structure from the *k + *1-th base. Other functions *g_m_*(·)*, m *= 4, . . . , 9) are irrelevant to 5ʹ - 3ʹ distance because their corresponding transitions for internal structures.

Total computational complexity using DFT with *U *parallel computing units, is *O*(*n*^4^*/U *) in time and *O*(*n*^2^*U *) in space (*S_max _*= *n *− 1*, α *= *β *= 1). In addition, since $Z_{i,j}^{b}$, $Z_{i,j}^{m}$, and $Z_{i,j}^{m1}$ do not contain variable *x*, therefore we can reduce the total amount of calculation by pre-computing them (See the Section S4 in the additional file 1).

### A distribution of 2D RNA folding landscapes

RNA2Dfold is an application for 2D projections of RNA folding landscapes which are the two-dimensional probability distributions whose axis correspond to the Hamming distances from two independent given reference structures [10]. Such distributions provide profound information on the whole ensemble through the medium of landscapes depending on the given structures. The RNA2Dfold, however, has difficulty of computational cost; it requires *O*(*n*^7^) in time and *O*(*n*^4^) in space though the computational time can be drastically improved by utilizing sparse matrices. On the other hand, extension of our proposed method reduces the complexity to less than *O*(*n*^5^) in time and *O*(*n*^2^) in space. Our method only calculate the distribution though RNA2Dfold also computes the minimum free energy structure of every combination of distances from the given structures. While a similar simplified algorithm has been proposed by [11], we construct an effective algorithm using DFT by expanding general principle described in previous sections.

#### Expanding the original model to two dimensions

The problem of computing the 2D folding landscape of an RNA, is defined as a natural expansion to two dimensions of the algorithm mentioned in the section. In this case, the objective distribution is defined on the two-dimensional discrete sample space which represents the Hamming distances from two given reference structures. Accordingly, we expand original model in equations (19)-(23) to two dimensions for the purpose of corresponding to two-dimensional score vectors. As shown in Algorithm S2, a vector variable **x **= (*x*_1_*, x*_2_) is employed to accumulate each component of a score vector **S **= (*S*_1_*, S*_2_) instead of applying a scalar variable *x*. The computational complexity of this model is *O*(*n*^3^*S*_1*max*_*S*_2*max*_*α*_1_*α*_2_*/U *) in time and *O*((*n*^2 ^+ *β*_1 _+ *β*_2_)*U *) in space, where *U *(≤ *S*_1*max*_*S*_2*max*_) is the number of parallel processing units, and *α_k _*and *β_k _*are time and space complexity for computing scoring function of *k*-th component.

#### Scoring functions

Now we can construct a model for the distribution of the Hamming distance from the two given structures by assigning *S*_1 _and *S*_2 _to the Hamming distance from the first and the second structures respectively. The total cost of this algorithm is *O*(*n*^3^*d*_1*max*_*d*_2*max*_*/U*) in time and *O*(*n*^2^*U*) in space. A concrete description is not shown here but in the Section S5 and S6 in the additional file 1.

### Run time evaluation and sample outputs

Next we show the run time of the above three algorithms. We adopt the minimum free energy (MFE) structures as the reference structures for the algorithms in the section 4.1 and 4.3. The other reference structure for the algorithm in the section 4.3 is the open chain structure, that is a completely no base pairing structure. We measured the run time by single or 8 cores, though theoretically we can distribute the process up to *S_max _*or *S*_1*max*_*S*_2*max*_.

**Figure 3:**
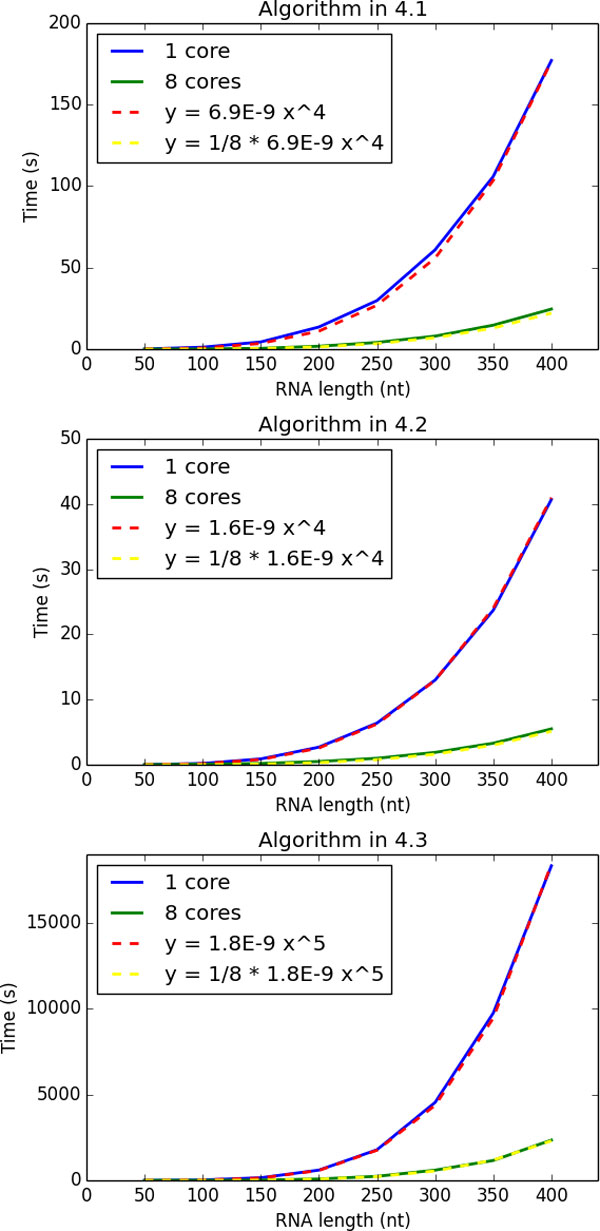
**Run time along the sequence lengths compared with theoretical curves**. Solid lines colored blue and green are run time measured as a mean of ten random sequences by single or eight cores respectively. Dashed lines are fitted curves theoretically expected.

As we can see in Figure 3, the curves of run time in each algorithms follow their theoretical orders, *O*(*n*^3^*dmax/U*), *O*(*n*^4^*/U*), and *O*(*n*^3^*d*_1*max*_*d*_2*max*_*/U*), where we consider *d_· _*to be proportional to RNA sequence length.

**Figure 4:**
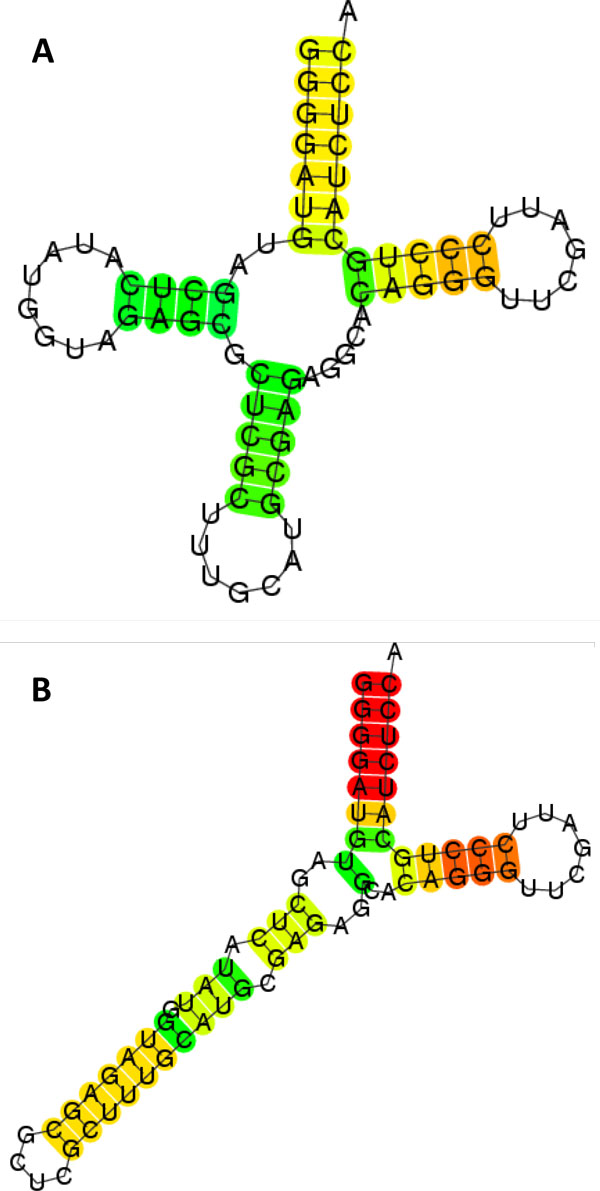
**Estimated structures of a tRNA sequences**. (a) A well-known cloverleaf structure, (b) a structure predicted by CentroidFold.

By way of example, we also illustrate outputs of our algorithms by using a sequence of tRNA. The secondary structure of tRNA is one of the most well-known structures called the cloverleaf structure (Figure 4(a)). However, prediction of the structure of a tRNA does not always have that shape. The CentroidFold [17], which is listed as one of the most accurate software tools in CompaRNA [18], predicts quite a different structure (Figure 4(b)). This disappointing example implies the limitation of RNA secondary structure predictions.

**Figure 5:**
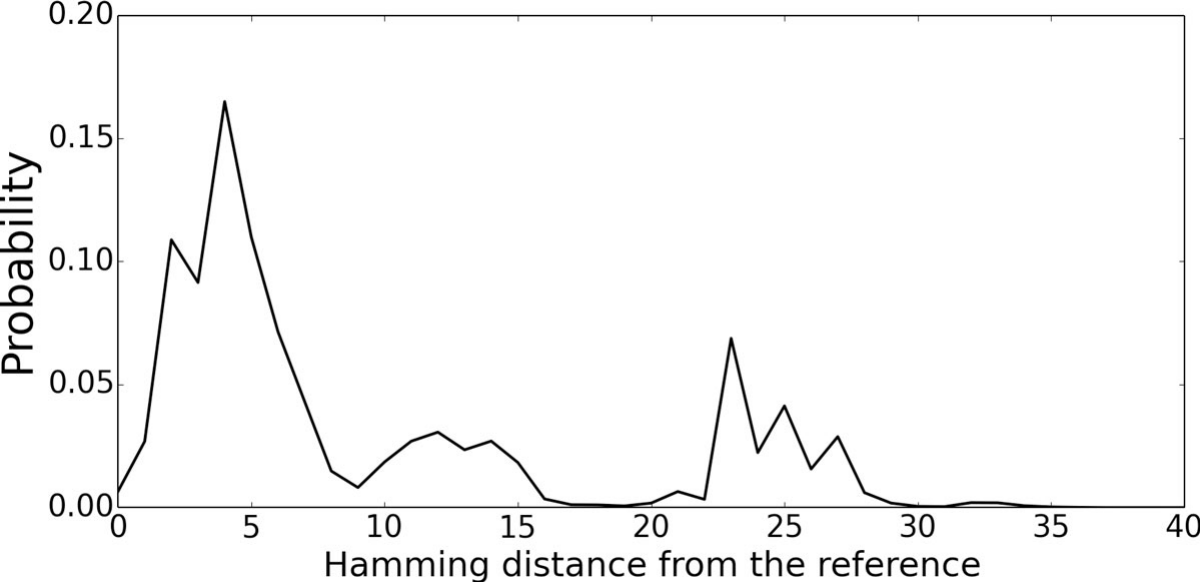
**The landscape of the probability distribution of Hamming distance**. X-axis represents the length of Hamming distance from the selected structure as a reference, and y-axis represents the probability that RNA folds into a structure which has each Hamming distance in the whole ensemble. We took a structure in Fig. 4(b) as the reference structure in this figure.

The probability distribution computed by the algorithm in the section 4.1 using the sequence and the reference structure illustrated in Figure 4(b) is shown in Figure 5. This probability landscape provides us an implication that this RNA might have sub-optimal structures around 25nt Hamming distance from the reference structure.

**Figure 6:**
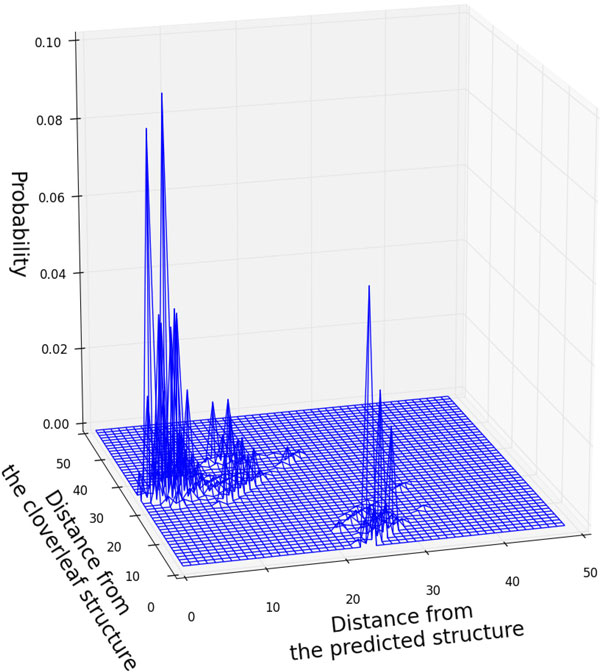
**2D expansion of tRNA structure existence probability landscape**. The landscape is drawn from the cloverleaf structure (Fig. 4(a)) and the predicted γ-centroid structure(Fig. 4(b)). We can see isolated population clusters around the both structures respectively.

The peak around 25nt in Figure 5, however, may not form a concrete sub-optimal cluster, because the peak is just the sum of the probabilities of the structures that have the similar Hamming distances around 25nt. The number of such structures is very large and those structures may distributed widely in the structure space because of the combinatorial explosion of base pairs (See the Section S7 in the additional file 1). In order to illustrate the distribution more precisely, we show in Figure 6, the 2D distribution computed by the algorithm in the section 4.3 using the cloverleaf structure (Figure 4(a)) and the CentroidFold structure (Figure 4(b)) as the references. In Figure 6 there seems to exit quite a high potential barrier between the CentroidFold structure and the cloverleaf structure. Although the biological reason why there is such a large structure cluster other than the cloverleaf structures remains unclear, it might be related to tRNA base modification, which is known to contribute to structure stability [19,20].

**Figure 7:**
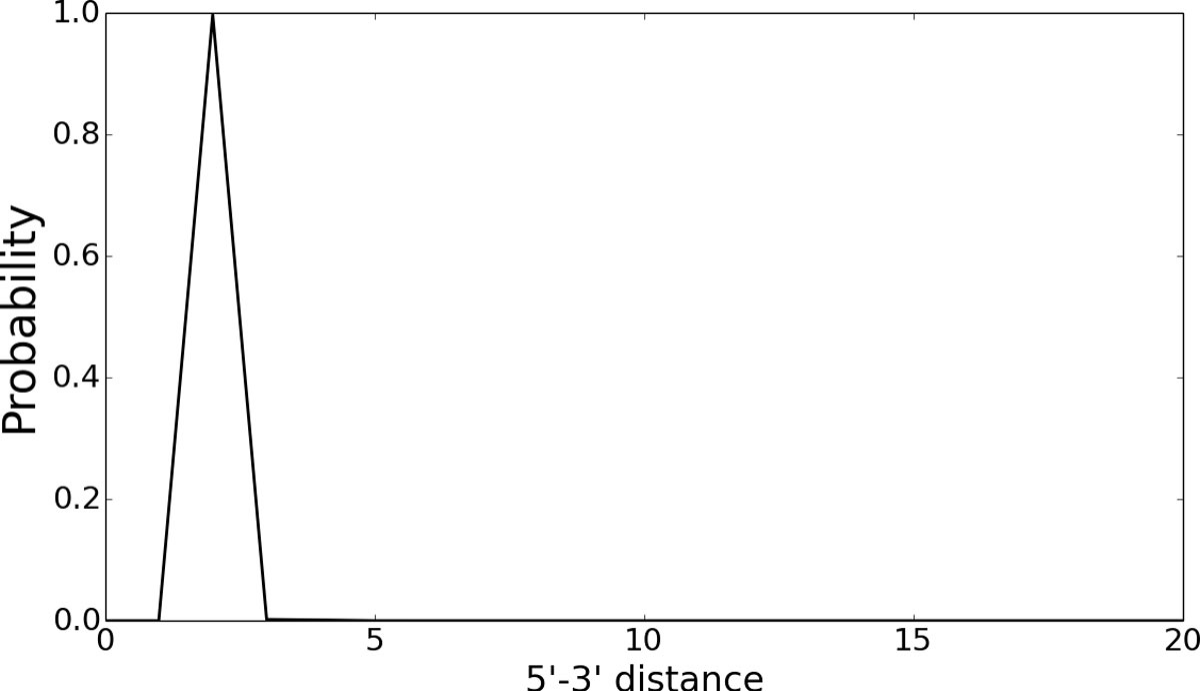
**The 5ʹ- 3ʹ distance distribution of the tRNA**. Although Fig. 6 implies this RNA can be fold into various structures, almost all the elements have the common feature from the point of view of 5ʹ-3ʹ distance.

We also draw a distribution of 5ʹ- 3ʹ distance for the tRNA sequence, which is obtained by the algorithm in the section 4.2 (Figure 7). We can see almost all the structures (more than 99.7%) have the same 5ʹ- 3ʹ distance although Figure 6 implies various structures are included in the ensemble. It indicates this tRNA is expected to fold into a certain compact structure near the 5ʹ- 3ʹ ends.

## Discussion and conclusions

Unreliable predictions of the RNA secondary structure have been prevented us from integrated analysis of RNA based on the estimated RNA structures. The energy model of the RNA secondary structure, however, offers rich information about the target RNA if we use appropriate algorithms to extract it. Such information is useful for analyzing not only the 3D structure prediction as a natural extension of secondary structure, but also the stabilities, the interactions with the other molecules, and so on.

In this paper, we proposed a general method to construct fast and accurate algorithms to compute the exact probability distributions of integer-valued features on the energy model of RNA secondary structures. We have shown that two useful algorithms, for Hamming distance from a reference structure and for 5ʹ- 3ʹ distance, can be constructed by just assigning the score functions *gk *(·). We extended the general method of an integer score to the method of an integer vector (2D), for the distributions of Hamming distances from two reference structures. We also applied those algorithms to tRNA as an example, and demonstrated the usefulness of observing the landscapes of probability distributions of the features. Although in some applications there have been proposed practically equivalent algorithms, the proposed method offers a clear and comprehensive guideline to design algorithms for a wide variety of integer features. The web server for the distributions of the Hamming distances is available at http://rtools.cbrc.jp/cgi-bin/index.cgi. We don't show the precise implementations for the other applications, but the proposed method is applicable to the integer features such as number of base pairs, or frequency of specific structure motifs by a little modification of original McCaskill model. In addition, distributions of combination of different integer scores can be also visualized by applying the 2D expanding technique described in the previous section.

## Competing interests

The authors declare that they have no competing interests.

## Authors' contributions

Ryota Mori constructed and implemented general and concrete algorithms, analyzed and interpreted the data, and drafted the article. Michiaki Hamada helped and advised Ryota Mori throughout the study especially when Ryota Mori confronted technical difficulties. Kiyoshi Asai designed the constitution of the article, headed the critical revision of the content, and gave final approval of the article.

## Supplementary Material

Additional file 1 ****Supplementary****.pdf. We explained the detail of our algorithms or a little ingenuity in this file. Click here for file

## Declarations

The publication charges for this work were funded by a Grant-in-Aid for Young Scientists (13J06668).

This article has been published as part of *BMC Genomics *Volume 15 Supplement 10, 2014: Proceedings of the 25th International Conference on Genome Informatics (GIW/ISCB-Asia): Genomics. The full contents of the supplement are available online at http://www.biomedcentral.com/bmcgenomics/supplements/15/S10.
