# Supplementary material for: Efficient calculation of exact probability distributions of integer features on RNA secondary structures
Source: BMC Genomics. 2014 Dec 12;15(Suppl 10):S6. doi: 10.1186/1471-2164-15-S10-S6 (PMC4304215; doi:10.1186/1471-2164-15-S10-S6)
Supplement: Additional file 1 — Supplementary.pdf. We explained the detail of our algorithms or a little ingenuity in this file. [file 1471-2164-15-S10-S6-S1.pdf]

Supplementary

S1 A description of the conventional model to obtain the partition function of RNA secondary structures

We show here an abstract form of computing the partition function.

Algorithm S1 An abstract form of calculating the partition function of RNA secondary structures

$Z[0] = 1$
for $k = 1$ to N **do**
    $Z[k] = \sum_{p=0}^{k-1} Z[p]t(k|p) + \sum_{p=0}^{k-2} \sum_{q=p+1}^{k-1} Z[p]Z[q]t(k|p,q) + c_k$
end for
$Z = Z[N]$

This procedure is quite similar to Algorithm 1 in our paper though we have additional terms in the recursion. As we can see in the McCaskill model, we need such calculations in the case of RNA secondary structures. We can construct Algorithm 3 from this by following the conversion of Algorithm 1 to Algorithm 2 in our paper.

S2 A method to obtain $g_k(\cdot)$ in constant time calculation for distributions of hamming distance from a reference structure

Firstly, we show full description of $g_k(\cdot)$.

$$g_1(i,j) = \sum_{p=i}^{j-1} \sum_{q=p+1}^{j} \mathcal{S}[p][q] \tag{S1}$$

$$g_2(i,j,k) = \sum_{p=i}^{k} \sum_{q=k+1}^{j} \mathcal{S}[p][q] \tag{S2}$$

$$g_3(i,j,k) = \sum_{q=k+1}^{j} \sum_{p=i}^{q-1} \mathcal{S}[p][q] \tag{S3}$$

$$g_4(i,j) = \sum_{p=i}^{j-1} \sum_{q=p+1}^{j} \mathcal{S}[p][q] + 1 - 2\mathcal{S}[i][j] \tag{S4}$$

$$g_5(i,j,k,l) = \sum_{p=i}^{k-1} \sum_{q=p+1}^{j} \mathcal{S}[p][q] + \sum_{p=k}^{l} \sum_{q=l+1}^{j} \mathcal{S}[p][q] + \sum_{p=l+1}^{j-1} \sum_{q=p+1}^{j} \mathcal{S}[p][q] + 1 - 2\mathcal{S}[i][j] \tag{S5}$$

$$g_6(i,j,k) = \sum_{p=k}^{j-1} \mathcal{S}[p][j] + \sum_{q=i+1}^{j} \mathcal{S}[i][q] + \sum_{p=i+1}^{k-1} \sum_{q=k}^{j} \mathcal{S}[p][q] + 1 - 2\mathcal{S}[i][j] \tag{S6}$$

$$g_7(i,j,k) = \sum_{p=i}^{k} \sum_{q=p+1}^{j} \mathcal{S}[p][q] \tag{S7}$$

$$g_8(i,j,k) = \sum_{p=i}^{k-1} \sum_{q=k}^{j} \mathcal{S}[p][q] \tag{S8}$$

$$g_9(i,j,k) = g_3(i,j,k). \tag{S9}$$

Equations (S1) - (S9) represent newly accumulated hamming distance from the reference structure by each transition.

They show that we need $O(n^2)$ calculation for $g_k(\cdot)$ which obtains hamming distance between two structures. This is one of the bottlenecks since these functions are embedded in recursive process. If we pre-calculate a vector C before the recursive process, we obtain $g_k(\cdot)$ by $O(1)$ calculation. Definition of

vector C corresponding to structure vector $\mathcal{S}$ is as follows:

$$C[i][j] = \sum_{k=i}^{j-1} \sum_{l=k+1}^{j} \mathcal{S}[k][l] \tag{S10}$$

Let us call it a cumulative structure vector.

C can be computed efficiently by dynamic programming technique:

Initialization:

$$C[i][i] = 0 \qquad (1 \leq i \leq n) \tag{S11}$$

$$C[i][i+1] = \mathcal{S}[i][i+1] \quad (1 \leq i \leq n-1) \tag{S12}$$

Recursion $(1 \leq i \leq n-1, i+1 < j \leq n)$ **:**

$$C[i][j] = \mathcal{S}[i][j] + C[i+1][j] + C[i][j-1] - C[i+1][j-1] \tag{S13}$$

This pre-calculation requires $O(n^2)$ time.

We wrote down $O(1)$ procedure for $g_k(\cdot)$ below:

$$g_1(i,j) = C[i][j] \tag{S14}$$

$$g_2(i,j,k) = C[i][j] - C[i][k] - C[k+1][j] \tag{S15}$$

$$g_3(i,j,k) = C[i][j] - C[i][k] \tag{S16}$$

$$g_4(i,j) = C[i][j] + 1 - 2\mathcal{S}[i][j] \tag{S17}$$

$$g_5(i,j,k,l) = C[i][j] - C[k][l] + 1 - 2\mathcal{S}[i][j] \tag{S18}$$

$$g_6(i,j,k) = C[i][j] - C[i+1][k-1] - C[k][j-1] + 1 - 2\mathcal{S}[i][j] \tag{S19}$$

$$g_7(i,j,k) = C[i][j] - C[k][j] \tag{S20}$$

$$g_8(i,j,k) = C[i][j] - C[i][k-1] - C[k][j] \tag{S21}$$

S3 Pre-calculating the maximum of distance between structures

We can find exact maximum value of hamming distance d_{max} though it never exceeds sequence length n:

$$d_{max} = \max_{\mathcal{S}_c \in \varsigma} \left[\sum_{i=1}^{n-1} \sum_{j=i+1}^{n} \mathcal{S}_r[i][j] \oplus \mathcal{S}_c[i][j] \right], \tag{S22}$$

where ς is a set of all possible candidate structure vectors and $\mathcal{S}_r$ is a reference structure vector.

We construct a $O(n^3)$ dynamic programming procedure so as to obtain d_{max}:

Initialization $(1 \leq i \leq n)$ **:**

$$D_{i,i} = D^1_{i,i} = D^b_{i,i} = D^m_{i,i} = D^m_{i,i-1} = 0 \tag{S23}$$

Recursion $(1 \leq i \leq n-1, i+1 < j \leq n)$ **:**

$$D_{i,j} = \max_k \left\{ \begin{array}{c} 0 \\ D_{i,k} + D^1_{k+1,j} - C[i][k] - C[k+1][j] \end{array} \right\} + C[i][j] \tag{S24}$$

$$D^1_{i,j} = \max_k \left\{ D^b_{i,k} - C[i][k] \right\} + C[i][j] \tag{S25}$$

$$D^b_{i,j} = \max_{k,l} \left\{ \begin{array}{c} 0 \\ D^b_{k,l} - C[k][l] \\ D^m_{i+1,k-1} + D^1_{k,j-1} - C[i+1][k-1] - C[k][j-1] \end{array} \right\} + C[i][j] - 2\mathcal{S}_r[i][j] + 1 \tag{S26}$$

$$D^m_{i,j} = \max_k \left\{ \begin{array}{c} D^m_{i,k-1} + D^1_{k,j} - C[i][k-1] - C[k][j] \\ D^1_{k,j} - C[k][j] \end{array} \right\} + C[i][j], \tag{S27}$$

where C is a vector which is defined in Supplementary section 0.1. We finally have d_{max} as $D_{1,n}$.

S4 A time-saving method for calculating $d_{5'-3'}$ exact distributions

We show a time-saving procedure for calculating $d_{5'-3'}$ exact distributions.

Algorithm S2 Exact calculation of a $d_{5'-3'}$ distribution by DFT approach

$O(n^3)$ pre-calculation for $Z^b_{i,j}, (1 \le i < j \le n)$ by McCaskill model
for $k = 1$ to $n-1$ **do**
  $x = \exp\left(2\pi i \frac{k-1}{n-1}\right)$
  $O(n^3)$ recursions for $Z_{1,n}[k]$ described on equations (S28) - (S31)
end for
for $k = 1$ to $n-1$ **do**
  $p(d_{5'-3'} = k) = \left\{\sum_{r=1}^{n-1} Z_{1,n}[r]\left(\cos\left(\frac{2\pi(r-1)}{n-1}\right) - i\sin\left(\frac{2\pi(r-1)}{n-1}\right)\right)\right\}/(n-1)$
end for

Recursions implied above are as follows:

Initialization $(1 \le i \le n)$ **:**

$$Z_{i,i}[k] = 1.0 \tag{S28}$$
$$Z^1_{i,i}[k] = 0 \tag{S29}$$

Recursion $(1 \le i < j \le n)$ **:**

$$Z_{i,j}[k] = x^{j-i} + \sum_{l=i}^{j-1} Z_{i,l}[k] Z^1_{l+1,j}[k] x \tag{S30}$$

$$Z^1_{i,j}[k] = \sum_{k=i+1}^{j} Z^b_{i,k} x^{j-k+1} \tag{S31}$$

S5 A framework of algorithm for the distribution of two-dimensional integer vector

In Algorithm S3, we show a method to expand our original algorithm to two dimensions.

Algorithm S3 2D expansion of the original model in Algorithm 4.

/* DP phase (distributed processing is available) */
for $S_1 = 0$ to S_{1max} **do**
  for $S_2 = 0$ to S_{2max} **do**
    $\mathbf{x} = \left(\exp\left[2\pi i \frac{S_1}{S_{1max}+1}\right], \exp\left[2\pi i \frac{S_2}{S_{2max}+1}\right]\right)$
    $Z[S_1][S_2][0] = 1$
    for $k = 1$ to N **do**
      $Z[S_1][S_2][k] = \sum_{p=0}^{k-1} Z[S_1][S_2][p] t(k|p) x_1^{s_1(p,k)} x_2^{s_2(p,k)} + \sum_{p=0}^{k-2} \sum_{q=p+1}^{k-1} Z[S_1][S_2][p] Z[S_1][S_2][q] t(k|p,q) x_1^{s_1(p,q,k)} x_2^{s_2(p,q,k)} + c_k x_1^{s_1(k)} x_2^{s_2(k)}$
    end for
    $\zeta_{S_1,S_2} = Z[S_1][S_2][N]$
  end for
end for
/* DFT phase*/
for $S_1 = 0$ to S_{1max} **do**
  for $S_2 = 0$ to S_{2max} **do**
    $z'_{S_1,S_2} = \left\{\sum_{r=0}^{S_{2max}} \zeta_{S_1,r} \exp\left[-2\pi i \frac{rS_2}{S_{2max}+1}\right]\right\} / (1 + S_{2max})$
  end for
end for
for $S_2 = 0$ to S_{2max} **do**
  for $S_1 = 0$ to S_{1max} **do**
    $z_{S_1,S_2} = \left\{\sum_{r=0}^{S_{1max}} z'_{r,S_2} \exp\left[-2\pi i \frac{rS_1}{S_{1max}+1}\right]\right\} / (1 + S_{1max})$
  end for
end for
$Z = \sum_{S_1=0}^{S_{1max}} \sum_{S_2=0}^{S_{2max}} z_{S_1,S_2}$

where $\mathbf{x} = (x_1, x_2)$, subscripts of 1 or 2 represent that they are variables or functions for the first and second components of a two-dimensional score vector respectively, and $P_{\mathbf{S}}$ means the probability that RNA sequence folds into a structure whose score is (S_1, S_2).

We have $p_{\mathbf{S}}$, the probability of obtaining score $\mathbf{S}$, by the following equation:

$$p_{\mathbf{S}} = \frac{z_{\mathbf{S}}}{Z} \tag{S32}$$

S6 A concrete description of recursions for the distribution of hamming distance from two reference structures

We show here concrete recursions to obtain the distribution of hamming distance from two reference structures. Naive expansion is quite simple; all we have to do is just exchanging $x^{g_k(\cdot)}$ for $x_1^{g_k^1(\cdot)} x_2^{g_k^2(\cdot)}$ such as:

$$Z_{i,j} = x_1^{g_1^1(i,j)} x_2^{g_1^2(i,j)} + \sum_{k=i}^{j-1} Z_{i,k} Z^1_{k+1,j} x_1^{g_2^1(i,j,k)} x_2^{g_2^2(i,j,k)} \tag{S33}$$

However, there are many meaningless calculations which can be cut by utilizing sparseness of the distribution. From constraints such as triangle inequality, we must satisfy the following expressions:

$$\forall \mathcal{S} \in \varsigma, \quad |d(\mathcal{S}_{R_1}, \mathcal{S}) - d(\mathcal{S}_{R_2}, \mathcal{S})| \leq d(\mathcal{S}_{R_1}, \mathcal{S}_{R_2}) \leq d(\mathcal{S}_{R_1}, \mathcal{S}) + d(\mathcal{S}_{R_2}, \mathcal{S}) \tag{S34}$$

$$\forall \mathcal{S} \in \varsigma, \quad d(\mathcal{S}_{R_1}, \mathcal{S}) \leq d_{1max} \tag{S35}$$

$$\forall \mathcal{S} \in \varsigma, \quad d(\mathcal{S}_{R_2}, \mathcal{S}) \leq d_{2max} \tag{S36}$$

$$\forall \mathcal{S} \in \varsigma, \quad \exists m \in \mathbb{N}, \quad d(\mathcal{S}_{R_1}, \mathcal{S}) + d(\mathcal{S}_{R_2}, \mathcal{S}) + d(\mathcal{S}_{R_1}, \mathcal{S}_{R_2}) = 2m, \tag{S37}$$

where $\mathbb{N}$ is a set of natural numbers, ς is a set of all possible secondary structure vectors, $\mathcal{S}_{R_i}(i = 1, 2)$ is a structure vector of i-th reference, and $d(\mathcal{S}_1, \mathcal{S}_2)$ means hamming distance between $\mathcal{S}_1$ and $\mathcal{S}_2$. Equation (S34) is derived from triangle inequality, and equation (S35) and (S36) are originated in definitions of d_{1max} and d_{2max}. A reason of equation (S37) is a little complicated. To put it simply, 1 bit transition of structure vector of $\mathcal{S}$ invariably causes 1 hamming distance changing from any other structures, and every structure vector can visit each other by repetition of 1 bit transitions.

We modified our algorithm to reduce meaningless calculation by converting axes. Abstract form is shown in Algorithm S4.

Algorithm S4 Improved 2D algorithm by DFT approach

$\delta = d(S_{R_1}, S_{R_2})$
$d'_{1max} = \delta$
$d'_{2max} = \frac{d_{1max}+d_{2max}-\delta}{2}$
for $d_1 = 0$ to d'_{1max} **do**
  for $d_2 = 0$ to d'_{2max} **do**
    $x = \exp\left(2\pi i \frac{d_1}{d'_{1max}+1}\right)$
    $y = \exp\left(2\pi i \frac{d_2}{d'_{2max}+1}\right)$
    $Z[d_1][d_2][0] = 1$
    for $k = 1$ to N **do**
      $Z[d_1][d_2][k] = c_k x^{s_x(k)} y^{s_y(k)} + \sum_{i=0}^{k-2} \sum_{j=i+1}^{k-1} Z[d_1][d_2][i] Z[d_1][d_2][j] t(k|i,j) x^{s_x(i,j,k)} y^{s_y(i,j,k)} + \sum_{i=0}^{k-1} Z[d_1][d_2][i] t(k|i) x^{s_x(i,k)} y^{s_y(i,k)}$
    end for
  end for
end for
for $d_1 = 0$ to d'_{1max} **do**
  for $d_2 = 0$ to d'_{2max} **do**
    $Z'[d_1][d_2] = \left\{\sum_{r=0}^{d'_{2max}} Z[d_1][r][N] \exp\left[-2\pi i \frac{r d_2}{d'_{2max}+1}\right]\right\} / (1 + d'_{2max})$
  end for
end for
for $d_2 = 0$ to d'_{2max} **do**
  for $d_1 = 0$ to d'_{1max} **do**
    $Z''[d_1][d_2] = \left\{\sum_{r=0}^{d'_{1max}} Z'[r][d_2] \exp\left[-2\pi i \frac{r d_1}{d'_{1max}+1}\right]\right\} / (1 + d'_{1max})$
  end for
end for
for $d_1 = 0$ to d_{1max} **do**
  for $d_2 = 0$ to d_{2max} **do**
    if $(|d_1 - d_2| \leq \delta \leq d_1 + d_2)$ and $(d_1 + d_2 + \delta$ is even$)$ **then**
      $p(d_1, d_2) = Z''[\frac{d_1-d_2+\delta}{2}][\frac{d_1+d_2-\delta}{2}]$
    else
      $p(d_1, d_2) = 0$
      /*where $p(d_1, d_2)$ is the probability that distance from structure 1 is d_1 and structure 2 is d_2*/
    end if
  end for
end for

In this case, we need a little modification to recursions as follows:

$$
\begin{aligned}
Z_{i,j} &= x^{\frac{g_1^1(i,j)-g_1^2(i,j)+\Delta\delta_1(i,j)}{2}} y^{\frac{g_1^1(i,j)+g_1^2(i,j)-\Delta\delta_1(i,j)}{2}} \\
&\quad + \sum_{k=i}^{j-1} Z_{i,k} Z^1_{k+1,j} x^{\frac{g_2^1(i,j,k)-g_2^2(i,j,k)+\Delta\delta_2(i,j,k)}{2}} y^{\frac{g_2^1(i,j,k)+g_2^2(i,j,k)-\Delta\delta_2(i,j,k)}{2}} \qquad \text{(S38)}
\end{aligned}
$$

$$
Z^1_{i,j} = \sum_{k=i+1}^{j} Z^b_{i,k} x^{\frac{g_3^1(i,j,k)-g_3^2(i,j,k)+\Delta\delta_3(i,j,k)}{2}} y^{\frac{g_3^1(i,j,k)+g_3^2(i,j,k)-\Delta\delta_3(i,j,k)}{2}} \qquad \text{(S39)}
$$

$$
\begin{aligned}
Z^b_{i,j} &= \sum_{k=i+2}^{j-1} Z^m_{i+1,k-1} Z^{m1}_{k,j-1} e^{-[f_3(i,j)/k_BT]} x^{\frac{g_6^1(i,j,k)-g_6^2(i,j,k)+\Delta\delta_5(i,j,k)}{2}} y^{\frac{g_6^1(i,j,k)+g_6^2(i,j,k)-\Delta\delta_6(i,j,k)}{2}} \\
&\quad + \sum_{k=i+1}^{j-2} \sum_{l=k+1}^{j-1} Z^b_{k,l} e^{-[f_2(i,j,k,l)/k_BT]} x^{\frac{g_5^1(i,j,k,l)-g_5^2(i,j,k,l)+\Delta\delta_4(i,j,k,l)}{2}} y^{\frac{g_5^1(i,j,k,l)+g_5^2(i,j,k,l)-\Delta\delta_5(i,j,k,l)}{2}} \\
&\quad + e^{-[f_1(i,j)/k_BT]} x^{\frac{g_4^1(i,j)-g_4^2(i,j)+\Delta\delta_1(i,j)}{2}} y^{\frac{g_4^1(i,j)+g_4^2(i,j)-\Delta\delta_4(i,j)}{2}} \qquad \text{(S40)}
\end{aligned}
$$

$$
\begin{aligned}
Z^m_{i,j} &= \sum_{k=i}^{j-1} \Bigg\{ e^{-[f_4(k-i)/k_BT]} x^{\frac{g_7^1(i,j,k)-g_7^2(i,j,k)+\Delta\delta_6(i,j,k)}{2}} y^{\frac{g_7^1(i,j,k)+g_7^2(i,j,k)-\Delta\delta_7(i,j,k)}{2}} \\
&\quad + Z^m_{i,k-1} x^{\frac{g_8^1(i,j,k)-g_8^2(i,j,k)+\Delta\delta_7(i,j,k)}{2}} y^{\frac{g_8^1(i,j,k)+g_8^2(i,j,k)-\Delta\delta_8(i,j,k)}{2}} \Bigg\} Z^{m1}_{k,j} \qquad \text{(S41)}
\end{aligned}
$$

$$
Z^{m1}_{i,j} = \sum_{k=i+1}^{j} Z^b_{i,k} e^{-[f_4(j-k)/k_BT]} x^{\frac{g_9^1(i,j,k)-g_3^2(i,j,k)+\Delta\delta_3(i,j,k)}{2}} y^{\frac{g_9^1(i,j,k)+g_9^2(i,j,k)-\Delta\delta_9(i,j,k)}{2}} \qquad \text{(S42)}
$$

where $g_k^i(\cdot)$ is $g_k(\cdot)$ whose reference structure vector is $\mathcal{S}_{R_i}$, and $\Delta\delta_k(\cdot)$ are:

$$
\begin{aligned}
\Delta\delta_1(i,j) &= E[i][j] && \text{(S43)} \\
\Delta\delta_2(i,j,k) &= E[i][j] - E[i][k] - E[k+1][j] && \text{(S44)} \\
\Delta\delta_3(i,j,k) &= E[i][j] - E[i][k] && \text{(S45)} \\
\Delta\delta_4(i,j) &= \Delta\delta_1(i,j) && \text{(S46)} \\
\Delta\delta_5(i,j,k,l) &= E[i][j] - E[k][l] && \text{(S47)} \\
\Delta\delta_6(i,j,k) &= E[i][j] - E[i+1][k-1] - E[k][j-1] && \text{(S48)} \\
\Delta\delta_7(i,j,k) &= E[i][j] - E[k][j] && \text{(S49)} \\
\Delta\delta_8(i,j,k) &= E[i][j] - E[i][k-1] - E[k][j] && \text{(S50)} \\
\Delta\delta_9(i,j,k) &= \Delta\delta_3(i,j,k) && \text{(S51)}
\end{aligned}
$$

$E[i][j]$ is defined by the hamming distance of partial structure vector:

$$E[i][j] = \sum_{p=i}^{j-1} \sum_{q=p+1}^{j} \mathcal{S}_{R_1}[p][q] \oplus \mathcal{S}_{R_2}[p][q] \tag{S52}$$

We have $E[i][j]$ effectively by the following recursions:

Initialization:

$$E[i][i] = 0 \qquad (1 \leq i \leq n) \tag{S53}$$

$$E[i][i+1] = \mathcal{S}_{R_1}[i][i+1] \oplus \mathcal{S}_{R_2}[i][i+1] \quad (1 \leq i \leq n-1) \tag{S54}$$

Recursion $(1 \leq i \leq n-1, i+1 < j \leq n)$ **:**

$$\begin{aligned} E[i][j] = \mathcal{S}_{R_1}[i][j] \oplus \mathcal{S}_{R_2}[i][j] + E[i+1][j] \\ +E[i][j-1] - E[i+1][j-1] \end{aligned} \tag{S55}$$

Meaning of $\Delta\delta_k(\cdot)$ is newly accumulated hamming distance between two reference structures by each transition, and it indicates the lower limit of $g_k^1(\cdot) + g_k^2(\cdot)$.

This formulation empirically contributes increase in speed several times though, of course, it depends on RNA sequence and reference structures. At least, we guarantee twice as fast as the original algorithm. Original and modified algorithms require $d_{1max}d_{2max}$ and $\frac{\delta(d_{1max}+d_{2max}-\delta)}{2}$ -time continuous calculations respectively. Thus, our modification contributes the following acceleration rate r:

$$r = \frac{d_{1max}d_{2max}}{\frac{\delta(d_{1max}+d_{2max}-\delta)}{2}} = \frac{2d_{1max}d_{2max}}{\delta(d_{1max}+d_{2max}-\delta)} \tag{S56}$$

r is a monotonic decrease function of δ from $\delta = 0$ to $\delta = \frac{d_{1max}+d_{2max}}{2}$:

$$\frac{\partial r}{\partial \delta} = \frac{2\delta - d_{1max} - d_{2max}}{(\delta(d_{1max}+d_{2max}-\delta))^2} \leq 0 \quad (0 < \delta \leq \tfrac{d_{1max}+d_{2max}}{2}) \tag{S57}$$

On the other hand, we have the following inequality from equations (S35) - (S36) and the property of arithmetic mean:

$$\delta \leq \min(d_{1max}, d_{2max}) \leq \frac{d_{1max}+d_{2max}}{2} \tag{S58}$$

Accordingly, we obtain the lower limit of r as follows:

$$\begin{aligned} r &= \frac{2d_{1max}d_{2max}}{\delta(d_{1max}+d_{2max}-\delta)} \\ &\geq \frac{2d_{1max}d_{2max}}{\min(d_{1max}, d_{2max})(d_{1max}+d_{2max}-\min(d_{1max}, d_{2max}))} = 2 \end{aligned} \tag{S59}$$

S7 Explosion of the number of possible structures

We have a massive number of possible structures with the distances from the reference structure. It is due to the combinatorial explosion of possible base pairs. Fig. S1 shows the number of structures of each Hamming distance from the reference.

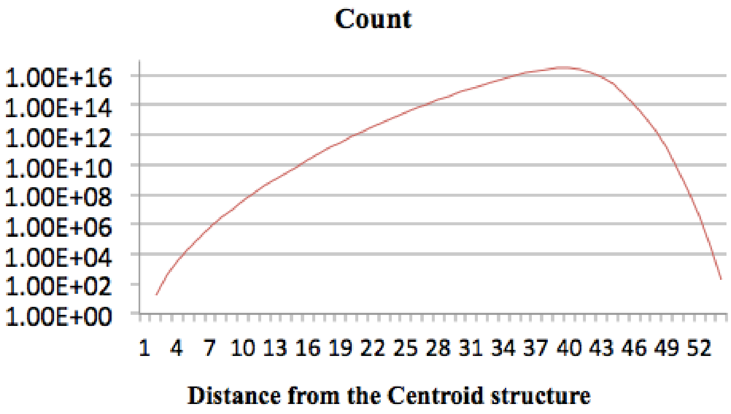

Figure S1: The number of possible structures
